# Live or death in cells: from micronutrition metabolism to cell fate

**DOI:** 10.3389/fcell.2023.1185989

**Published:** 2023-05-12

**Authors:** Yuting Wang, Wei Wu, Jianke Gong

**Affiliations:** ^1^ Key Laboratory of Molecular Biophysics of MOE, College of Life Science and Technology, Huazhong University of Science and Technology, Wuhan, Hubei, China; ^2^ State Key Laboratory for Diagnosis and Treatment of Infectious Diseases, National Clinical Research Center for Infectious Diseases, The First Affiliated Hospital, College of Medicine, Zhejiang University, Hangzhou, China

**Keywords:** cell death, nutrition, metabolism disorder, signal pathway, *Caenorhabditis elegans*

## Abstract

Micronutrients and cell death have a strong relationship and both are essential for human to maintain good body health. Dysregulation of any micronutrients causes metabolic or chronic diseases, including obesity, cardiometabolic condition, neurodegeneration, and cancer. The nematode *Caenorhabditis elegans* is an ideal genetic organism for researching the mechanisms of micronutrients in metabolism, healthspan, and lifespan. For example, *C. elegans* is a haem auxotroph, and the research of this special haem trafficking pathway contributes important reference to mammal study. Also, *C. elegans* characteristics including anatomy simply, clear cell lineage, well-defined genetics, and easily differentiated cell forms make it a powerful tool for studying the mechanisms of cell death including apoptosis, necrosis, autophagy, and ferroptosis. Here, we describe the understanding of micronutrient metabolism currently and also sort out the fundamental mechanisms of different kinds of cell death. A thorough understanding of these physiological processes not only builds a foundation for developing better treatments for various micronutrient disorders but also provides key insights into human health and aging.

## Introduction

**FIGURE 1:**
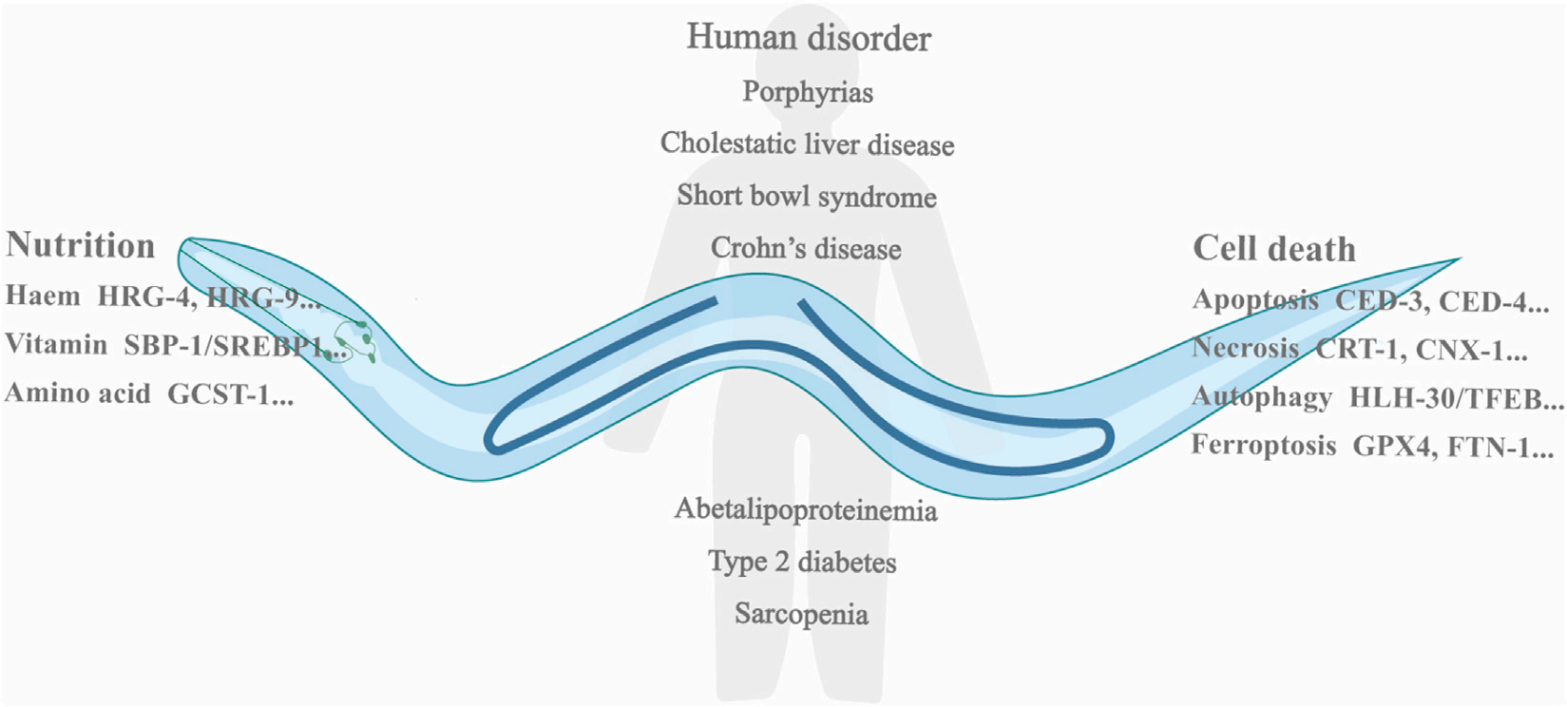
Showing micronutrient limitation related disorders in human and listing the genes in different types of nutrition metabolism and cell death in *C. elegans*. Nutrient deficiency causes multiple metabolism disorders in human, including porphyrias, cholestatic liver disease, short bowl syndrome, Crohm’s disease, and sarcopenia. In *C. elegans*, many genes are involved in the metabolisms of micronutrient, like HRG-4, HRG-9, SBP-1, and glycine cleavage system T-protein (GCST-1). Meanwhile, different kinds of cell death occur in *C. elegans*, and essential genes are also listed, for example, CED-3, CED-4, HLH-30/TFEB, and FTN-1.

Nutrients are the foundations of organisms’ activities and survival. In addition to macromolecules carbohydrates, lipids, and proteins, many micronutrients including haem, vitamin, and amino acid are focused as essential elements of life. These micronutrients are principles to support biosynthetic and bioenergetic processes for growth, development, healthspan, and lifespan. Deficiency of micronutrients causes metabolic disorders and health damage (Figure 1). Organisms as seemingly different as nematode and mammals have much in common regarding nutrition metabolism. In *C. elegans*, different kinds of cell death are beneficial to overcome nutrient-limiting or stress conditions. For instance, vitamin B12 deficiency during early-life worms causes ferroptosis in germ-cell (Qin et al., 2022). Threonine supplementation protects against age-associated ferroptosis through the DAF-16 and HSF-1 pathways (Kim et al., 2022). Indeed, *C. elegans* is used as a genetic and visual model for cell death research due to easily observable morphology of cell death in living animals. Cell death is regulated by complex mechanisms during different cellular stages and research *in vitro* neglects many details of cell death. Studying cell death *in vivo* is more advantageous in *C. elegans*. Notably, regulation mechanism of cell death in *C. elegans* is similar to mammals. In this review, we discuss the essential molecule and regulatory pathway. It is hoped the researches on nutrition disorders and cell fate provide different understanding.

### The metabolism of haem

Haem is considered the essential biomolecule because of transferring electrons, binding oxygen, and catalyzing redox reactions with it in cellular (Koreny et al., 2022). Besides being used as an element of the electron transport chains, haem plays a vital function in the anabolic metabolism of steroids, fats, and further secondary metabolites, as well as the catabolism of exogenous substrates and endogenous metabolic complexes (Schenkman and Jansson, 2003). As a component of haemoglobin or myoglobin, haem is responsible for transporting diatomic gases to the blood in mammals (Chen et al., 2008). Haem in signal transduction, a sensor of nitric oxide (NO), is binding NO to the haem-containing domain, and subsequently soluble guanylyl cyclase is activated by the cyclase domain (Montfort et al., 2017). Haem plays a crucial regulatory molecule modulating the function of binding proteins like transcription factors and ion channels (Hou et al., 2006). The via of biosynthesizing haem is conserved, multi-step enzymatic pathway using amino acid, cofactors, and substance ferrous iron (Phillips, 2019). In the biosynthesis of haem, the initial and final three reactions happen in the mitochondria, while remaining steps take place in the cytosol (Koreny et al., 2022). The intermediates traverse organelle membranes for haem biosynthesis to proceed (Schultz et al., 2010). When haem biosynthetic pathway is defective, pathway intermediates are accumulated, which causes porphyrias. Porphyrias, a generic title for a type of metabolic disorder, leads to either neurologic or mitochondrial membrane photocutaneous symptoms phenotypically based on the haem metabolic intermediate that accumulates (Phillips, 2019).

**FIGURE 2:**
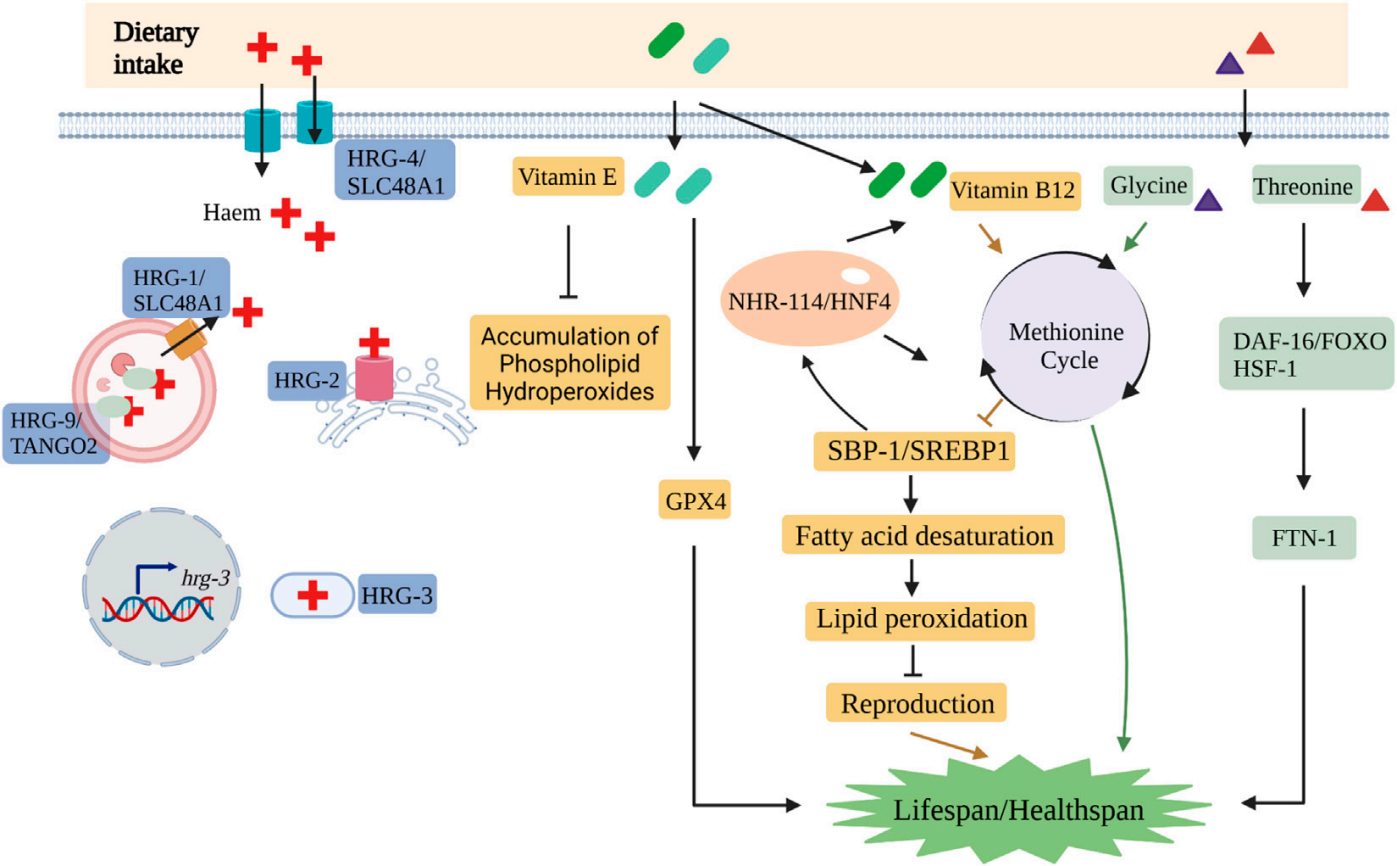
The nutrient metabolisms include many critical pathways in *C. elegans*. The key molecules involved in the trafficking pathways of haem are HRG-1/SLC48A1, HRG-2, HRG-3, HRG-4/SLC48A1 and HRG-9/TANGO2.1 in *C. elegans*. Vitamin B12 affects reproduction by methionine cycle-SBP-1/SREBP1-lipogenesis axis. Vitamin E suppresses the accumulation of phospholipid hydroperoxides and extends the lifespan of *C. elegans* via GPX4. Glycine and threonine play important roles in the lifespan and lipid peroxidation, respectively. (Created with BioRender.com).

Haem cannot be biosynthesized by *C. elegans*, so it is a favorable organism to research haem transferring pathways (Rao et al., 2005). In *C. elegans*, intestinal cells are responsible for the assimilation of haem exclusively from peripheral environment. Besides intaking haem from the diet and trafficking it to various tissue locations, *C. elegans* intestinal cell is involved in storing haem (Rajagopal et al., 2008; Chen et al., 2011; Korolnek et al., 2014). Haem-responsive gene 9 (HRG-9) in *C. elegans*, a haem chaperone, is conserved compared with yeasts and zebrafish and plays a crucial role in trafficking haem from its storage conditions to other tissue (Sun et al., 2022). HRG-4 traffics haem into worm intestinal cells, while HRG-1 is involved in mediating haem homeostasis in intracellular conditions (Rajagopal et al., 2008). HRG-3, a haem-trafficking protein, is responsible for intracellular carrier, but also for tissue-to-tissue transfer, including intestine-to-hypodermis, intestine-to-oocytes, intestine-to-muscle, intestine-to-neurons (Chen et al., 2011; Korolnek et al., 2014). Under HRG-3 deficiency, haem transport is dysregulated, which leads to either embryonic lethality or development arrest after hatching (Chen et al., 2011) (Figure 2).

### The metabolism of vitamin

An organism always intakes diet which are converted available substances for reproduction, cell regeneration, and wound repair, as well as energy to support daily cellular and organismal function. Micronutrients including vitamins, cofactors, and essential minerals indirectly contribute to the generation of biomass and energy (Yilmaz and Walhout, 2014). Vitamin is an essential micronutrient for health that is much less abundant. Many vitamins are not synthesized by human or animal themselves and hence need to be obtained from daily diet. Recent studies revealed that body development and health were negatively affected because of vitamin deficiency. For instance, the current studies focus on the impact of maternal vitamin B12 deficiency during developmental offspring, specifically, it also causes the increase of obesity and insulin resistance in subsequent generations (Yajnik et al., 2008; Stewart et al., 2011; Ghosh et al., 2017; Tanwar et al., 2020). Vitamin C deficiency severely leads to scurvy, a potentially fatal condition (Carr and McCall, 2017). Vitamin D insufficiency is associated with autoimmune diseases, hypertension, diabetes, and cancer, particularly chronic kidney disease (Holick, 2005; Holick, 2007; Chau and Kumar, 2012). Shortage of vitamin E is linked to intestinal fat absorption diseases in human, such as hepatic cholestasis, short bowl syndrome, and Crohn’s disease (Muller et al., 1985). Vitamin supplementations with pharmacologic doses are beneficial for a healthy lifecycle in all kinds of organisms.

Because *C. elegans* lifespan is about 2–3 weeks at 20°C, it is an ideal model organism to systematically examine how vitamin affects longevity. *C. elegans* absorbs micronutrients by diet for growth and development. Depletion of specific vitamins induces developmental slowing or developmental arrest. For example, the study has revealed that vitamin B12 insufficiency during early-life worms occurred fat accumulation and reactive oxygen species (ROS) production in mature worms that subsequently triggered germline disorders due to ferroptosis (Qin et al., 2022). When vitamin B12 deficiency happened in the early life of worms, adult worms’ characteristics were affected by the methionine circuit, SBP-1/SREBP1 activity, and synthesis of lipid (Qin et al., 2022). Additionally, SBP-1/SREBP1 is involved in crucial feedback metabolism to regulate organismal vitamin B12 homeostasis (Qin et al., 2022). In *C. elegans*, administration of vitamin E not only suppressed the accumulation of phospholipid hydroperoxides but also prolonged the lifespan of mutant that lacks all homologous genes of GPX4 (Sakamoto et al., 2020). Moreover, intake of vitamin E during reproductive stage extended the *C. elegans* lifespan significantly (Sakamoto et al., 2020) (Figure 2).

### The metabolism of nutrient amino acid

In the studies of model animals, dietary restriction (DR) refers to the targeted decrease of dietary components not including vitamins and minerals, and has been shown to promote longevity and improve adult worms’ healthspan (Green et al., 2022). Amino acid metabolism sustains organism life by serving as protein subunit, energy substrate, glutathione, and neurotransmitters. Disorder of nutrient amino acid has been studied and found to have serious effects on many physical functions, like maintaining healthspan and the modulation of aging. Leucine is a pharmaconutrient. In type 2 diabetes and sarcopenia disease, leucine supplementation has primarily been evaluated as a potential factor (van Loon, 2012). Leucine stimulates release of insulin and suppresses muscle protein damage and biosynthesis (van Loon, 2012). Administration of glycine from dietary supplementation is effective in treatment of metabolic disorders cardiovascular diseases, inflammatory diseases, obesity, diabetes, and cancer (Razak et al., 2017). Dysregulation of methionine metabolism is also associated with chronic liver diseases (Li et al., 2020a).

In *C. elegans*, many researchers have concentrated on the connection between nutrient amino acid supply and the regulation of longevity. Glycine extended the lifespan by the effect of methionine cycle in *C. elegans* (Liu et al., 2018). In aging *C. elegans*, glycine is accumulated steadily and significantly because the expression level of glycine cleavage and purine synthesis pathway genes decrease (Liu et al., 2018). However, by supplementing with glycine in diet, the lifespan in *C. elegans* is rescued with mutations in two methionine synthase enzymes, *metr-1*(*ok521*) and *sams-1*(*ok3033*) (Liu et al., 2018). The worms *daf-2(e1370)* and *eat-2(ad456)* are long-lived phenotypes, where glycine mediated longevity via the methionine cycle-dependent manner, and all of these findings remain consistent with serine supplementation (Liu et al., 2018). A biguanide drug metformin is extensively treated for type 2 diabetes and metabolic syndrome. The impact of metformin is mediated by *E. coli* that is co-cultured with it, with metformin inhibiting folate production and methionine circuit of *E. coli*. Consequently, the changed methionine metabolism induced by the co-cultured *E. coli* extended *C. elegans* lifespan (Cabreiro et al., 2013). A responsive complex via the methylglyoxal-regulated proteasome is activated by disturbed threonine catabolism, which further enhanced healthspan of *C. elegans* (Kim et al., 2022). The longevity effects of threonine rely on cellular oxidative stress and transcription factors *daf-16* and *hsf-1* (Kim et al., 2022) (Figure 2).

### The mechanism of apoptosis

Apoptosis is one of programmed cell death and is critically important for genome stability, stress response, immunity, development, and differentiation (Fuchs and Steller, 2011; Kuranaga, 2011). Apoptosis is conserved across the evolutionary tree from lower creatures to mammals. In mammals, apoptosis is induced by the intrinsic and extrinsic pathways (Ow et al., 2008). Among metazoans, the intrinsic pathway is highly conserved. However, the critical stage from mitochondria, the release of cytochrome C, is not detected in *C. elegans* and *Drosophila* (Oberst et al., 2008). In apoptosis, the transcriptional level of BH3 (one of the BCL-2 family), and release of cytochrome C, is increased by intrinsic controlled and genotoxic agents within mitochondria. Caspase 9 is activated by combination of cytochrome C and Apaf-1, which further causes the activation of caspases 3 and 7 (Oberst et al., 2008). The fas ligand (FasL), tumor necrosis factor (TNF), and TNF-related apoptosis-inducing ligand (TRAIL) are all involved in activating the extrinsic death pathway (Nagata, 1997; Krammer, 2000; Strasser et al., 2009). When FasL and its receptor Fas are combined, it triggers the generation of death-inducing signaling complex (DISC) (Oberst et al., 2008). The complex is composed of Fas, called FADD (an adaptor protein), and procaspase 8 (Oberst et al., 2008). The DISC generation results in the activation of caspase 8, which subsequently triggers two different cell types. One type (like thymocytes), caspase 8 directly triggers caspase 3 to lead to cell death. Another type (like hepatocytes), caspase 8 cleaves the protein Bid, which is the BCL-2 family. Bid promotes to increase of cytochrome C, which further activates caspase 9 and caspase 3.

**FIGURE 3:**
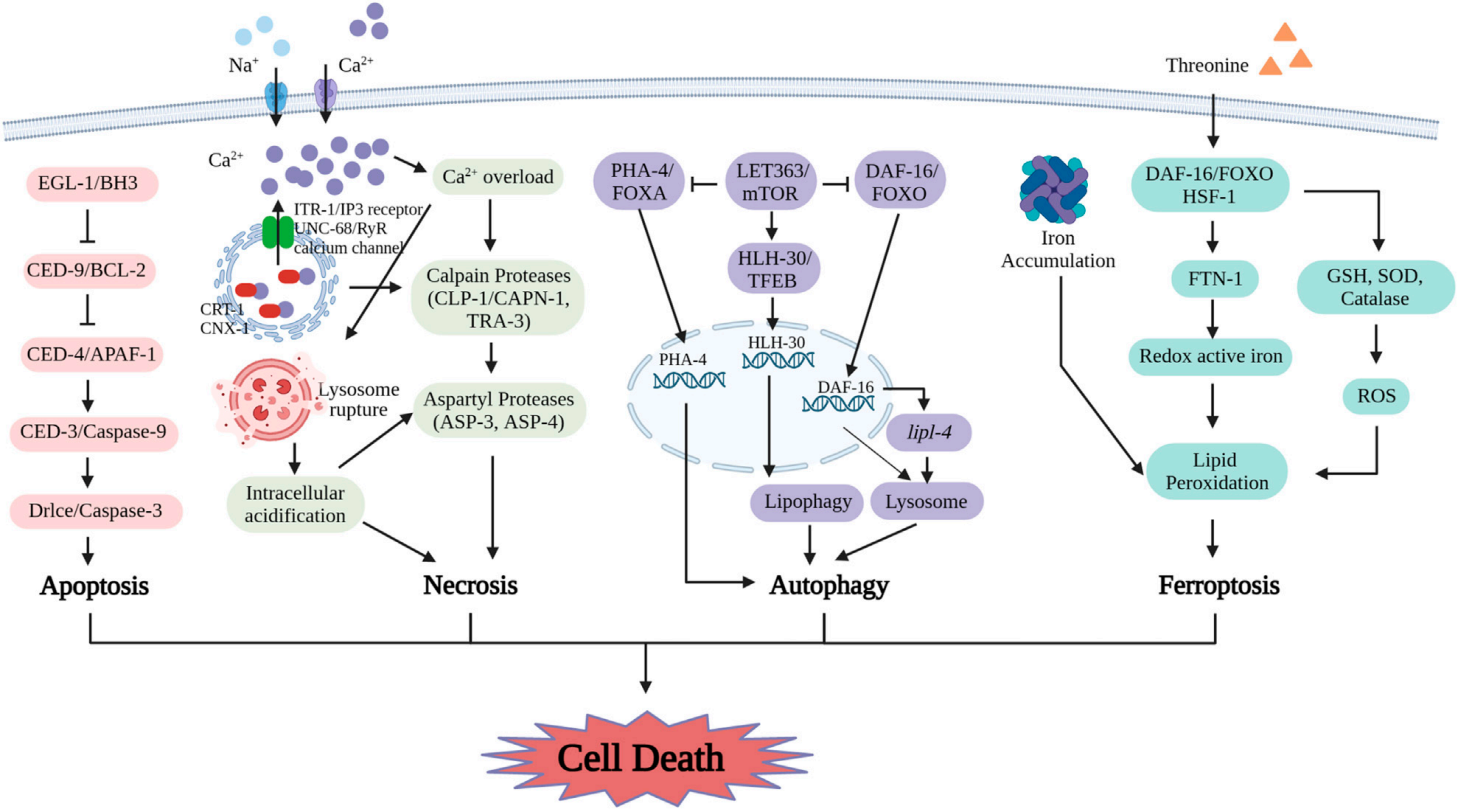
The mechanisms of cell death including apoptosis, necrosis, autophagy and ferroptosis in *C. elegans* are uncovered with key detail. The proteins involved in apoptosis are EGL-1/BH3, CED-9/BCL-2, CED-4/APAF-1, CED-3/Caspase-9 and Drlce/Caspase-3. In necrosis, these molecules include ITR-1/IP3 receptor, UNC-68/RyR calcium channel, CRT-1, CNX-1, CLP-1/CAPN-1, TRA-3, ASP-3 and ASP-4. The key regulators in autophagy include LET363/mTOR, PHA-4/FOXA, DAF-16/FOXO and HLH-30/TFEB. Ferroptosis process is induced by ferritin-dependent manner. Supplement of threonine affects ferroptosis by lipid peroxidation including the levels of DAF-16/FOXO, FTN-1, redox active iron, GSH, SOD, catalase and ROS. (Created with BioRender.com).

In *C. elegans*, apoptosis takes place in embryonic development and adult germline (Conradt et al., 2016). In 2002, Sydney Brenner, John E. Sulston, and H. Robert Horvitz received the Nobel Prize for Medicine because of significant contributions to discovering the cell lineage and identifying the molecular mechanism of cell death in *C. elegans*. The main proteins playing a vital role in apoptosis in *C. elegans* are EGL-1 and CED-family members (Adams and Cory, 2001) (Figure 3). EGL-1 is a crucial activator of apoptotic cell that is destined to die. CED-9 plays an important role like BCL-2 in preventing apoptosis (Adams and Cory, 2001). CED-4 (homolog of APAF-1 in mammalian) functions as a trigger for CED-3, leading to the initiation of cell death (Gartner et al., 2008). Elevated activity of EGL-1 blocks the activity of CED-9, activating CED-4 and CED-3, which ultimately triggers the process of apoptosis (Horvitz, 2003; Gartner et al., 2008; Conradt et al., 2016). CED-3, CED-4, EGL-1, and CED-9 are all participated in a pathway in that EGL-1 initiates cell death by acting upstream of CED-9, CED-9 prevents cell death by acting upstream of CED-4, and CED-4 activities cell death via upstream of CED-3 (Aballay and Ausubel, 2001). CED-8 acts as a substrate for CED-3 caspase cleavage, which induces apoptosis (Chen et al., 2013; Suzuki et al., 2013).

### The mechanism of necrosis

Necrosis, a form of programmed cell death, frequently occurs in severe environments (Walker et al., 1988). Characteristics of necrosis are cellular and organelle swelling, breakdown of the premature membrane, and the release of intracellular substances to surrounding tissues, which leads to inflammation (Majno and Joris, 1995; Festjens et al., 2006).

The initial molecular mediator of necrosis pathway was cyclophilin D, a positive regulator that modulates the opening probability of the mitochondrial permeability transition pore (mPTP) (Baines et al., 2005; Halestrap, 2009). The initiation of necrosis via the mitochondrial pathway is triggered by the activation of mPTP in the inner mitochondrial membrane (IMM), which is activated by Ca^2+^ influx. In necrosis, the rapid activation of the mPTP leads to the dissipation of the proton that is responsible for driving ATP biosynthesis in mitochondrial across the IMM (Del Re et al., 2019). The mPTP opening by Ca^2+^ induced in the mitochondrial pathway plays a crucial role during necrosis. Another form of necrosis is affected by apoptosis receptors as well as the receptor-interacting protein kinases 1 and 3 (RIPK1 and RIPK3) (Galluzzi et al., 2009). In the death receptor pathway, the activation of RIPK3, a serine/threonine kinase, is the key event that triggers necroptosis, which is carried out through phosphorylation by its homologous RIPK1. Once activated, RIPK3 phosphorylates and a pseudokinase called mixed lineage kinase-like domain (MLKL), which oligomerizes and permeabilizes the plasma membrane, resulting in necrosis. RIPK3-MLKL pathway is recognized as the specific mechanism during necrosis (Del Re et al., 2019). Another type of induced necrosis occurs when poly ADP-ribose polymerase (PARP) is excessively triggered due to DNA damage (Zong et al., 2004). PARP1 is a nuclear enzyme, which is quickly triggered by the break of DNA and induces DNA restoration by adding ADP-ribose to chromatin-correlated proteins (Degterev and Yuan, 2008). Alkylating DNA damage induces to decrease of NAD^+^ by PARP1 in cytosolic that results in necrosis via ‘energy collapse’ during glycolysis that relies on cytosolic NAD^+^ for energy metabolism (Zong et al., 2004).

In *C. elegans*, the mechanisms and morphological features of necrosis resulting from aberration channel function and ionic imbalance exhibit similarities to excitotoxicity observed in vertebrates (Nikoletopoulou and Tavernarakis, 2014). Previous research discussed a novel example for excitotoxicity by examining the systemic effects of various triggers on glutamate-induced necrosis (Mano and Driscoll, 2009). Excitotoxicity critically depends on Ca^2+^ influx through glutamate-gated ion channels. Intracellular calcium overload is considered one of the initial steps in the necrosis pathway (Kourtis and Tavernarakis, 2007). In the necrosis pathway, endoplasmic reticulum Ca^2+^ binding proteins (CRT-1 and CNX-1) and channels (UNC-68 and ITR-1), Ca^2+^ dependent proteases (CLP-1 and TRA-3), and mitochondria permeability transition (MPT) are involved in regulating Ca^2+^ signaling (Geng et al., 2012). Gain-of-function mutations *mec-4*, the DEG/ENaC subunit, activate six mechanosensory neurons to result in excitotoxic necrosis of the cells in *C. elegans* (Syntichaki and Tavernarakis, 2003; Furuta et al., 2021). The gain-of-function *mec-4* mutation alters the generation of the mechanosensory Na^+^ channel and makes it permeable to extracellular Ca^2+^ (Bianchi et al., 2004). The mutant *mec-4* also causes necrosis and is partially suppressed by calreticulin. The Ca^2+^ chaperone residing in the endoplasmic reticulum (ER) assists in accumulating Ca^2+^ within the ER while deactivating channels that release Ca^2+^ from the ERs (Xu et al., 2001). Intracellular pH is a crucial inducing factor of necrosis in *C. elegans*. During necrosis, the vacuolar H^+^-ATPase is a pump that is responsible for acidifying lysosomes and other organelles within cell is necessary to promote necrosis after cytoplasmic calcium overload (Syntichaki et al., 2005). In necrosis, overload calcium actives calpain, which disrupted lysosomal membranes leading to the increase of acidic contents in cytoplasm (Syntichaki and Tavernarakis, 2003; Syntichaki et al., 2005) (Figure 3).

### The mechanism of autophagy

Autophagy is an evolutionarily conserved cell death in that intracellular macromolecules are decomposed into multiple substances in the lysosomes (Mizushima and Komatsu, 2011). The main classifications of autophagy are macroautophagy, microautophagy, and chaperone-mediated autophagy. Macroautophagy is the most extensive type of autophagy compared to microautophagy and chaperone-mediated autophagy. The crucial functions of autophagy are to eliminate impaired or aged organelles and sustain energy homeostasis and engage intracellular nutrients to satisfy energy demands during nutrient deficiency (Klionsky and Emr, 2000).

Autophagy is strongly activated by nutrient deficiency and multiple stresses and quickly produces autophagosomes (Mizushima and Komatsu, 2011). Autophagy is activated by dephosphorylation of the serine or threonine protein kinase (ULK1). The ULK1 complex, consisting ULK1, ATG13, ATG101, and FIP200, breaks up from mechanistic/mammalian target of rapamycin complex 1(mTORC1) complex. The initiation of autophagy is activated by ULK1 and the phosphorylates ATG13 and FIP200 (Chang and Neufeld, 2009; Hosokawa et al., 2009; Jung et al., 2009). Phosphorylation of both Ambra1 and Beclin-1 by ULK1 complex results in the activation of the Beclin-1-Vps34 complex (Beclin-1, Vps34, Vps15, and ATG14L) (Di Bartolomeo et al., 2010). Autophagy is triggered by the interplay of Ambra1 and TRAF6, which modulates the balance and activity of ULK1 (Nazio et al., 2013). Autophagy is also induced through phosphorylation of Beclin-1 by AKT and EGFR1 (Wang et al., 2012; Wei et al., 2013). Autophagosome generation is enhanced by the recruitment of effector proteins through phosphatidylinositol-3-phosphate (PI3P) produced by Vps34 (Axe et al., 2008; Polson et al., 2010; Lu et al., 2011). The extension of the autophagosome is promoted by two systems, ATG12–ATG5–ATG16L and LC3–phosphatidylethanolamine (Ichimura et al., 2000; Kuma et al., 2002; Fujita et al., 2008). After the establishment of fully developed autophagic vesicles, mature autophagosomes, and lysosomes together merge, resulting in autolysosomes responsible for the decomposition of molecules and organelles (Mizushima and Komatsu, 2011).

In *C. elegans*, autophagy occurs mainly due to dietary restrictions and stress conditions. In *C. elegans*, initiation of autophagy is modulated by critical elements, such as mTOR signaling, a serine/threonine kinase, and DAF-16(a homolog of mammalian FOXO) that acting as an activator (Dall and Færgeman, 2019) (Figure 3). Both dietary restriction and suppression of the mTOR pathway activate autophagy in *C. elegans*. Conversely, inactivation of autophagy-related genes prevents the lifespan extension caused by these conditions (Hansen et al., 2008). Extended lifespan due to LET-363, DAF-15, or RSKS-1 mutant was eliminated by the autophagy modulatory transcription factor HLH30/TFEB and autophagy proteins (Hansen et al., 2008; Lapierre et al., 2013). *let-363* or *rsks-1* RNAi results in extending lifespan, which requires the involvement of PHA-4, which manages autophagy and is counteracted by mTORC1 pathway (Sheaffer et al., 2008). Autophagy has beneficial effects on metabolism. Autophagy is activated by upregulated transcriptionally of lysosomal acid lipase *lipl-4*, which extends lifespan via lipid regulatory (Lapierre et al., 2011; Folick et al., 2017). LGG-1 and LGG-2, are two ATG8 orthologs in *C. elegans* that function to degrade sequestosome-related (SQST-1) at different stages of the aggrephagy pathway (Wu et al., 2015). LGG-1 is necessary for the disassembly of SQST-1 throughout development, whereas the necessity for LGG-2 is temporal (Wu et al., 2015). In the embryonic period, a degradation defect of SQST-1 aggregates occurs when LGG-2 disables its role, whereas in the larval, SQST-1 is eliminated as normal (Wu et al., 2015). Autophagy mutants show distinct models of SQST-1 aggregation in various tissues and different period (Zheng et al., 2020). Different autophagy mutants exhibit distinct modes of SQST-1 aggregates in various tissues and periods (Zheng et al., 2020). Calpain family member CLP-2 degrades SQST-1 in a tissue- and stage-specific way in *C. elegans* (Zheng et al., 2020). Regulation of lipid phase separation of PGL particles by mTORC1 signaling under heat stress during embryonic period contributes to coordinating heat stress adaptation and autophagic degradation (Zhang et al., 2018a).

### The mechanism of ferroptosis

Ferroptosis is a form of cell death, which depends on iron and is distinguished by oxidative injury to cellular membranes (Dixon et al., 2012; Galluzzi et al., 2018). The occurrence of ferroptosis has been established in diverse mammals, such as murine and cancer cell lines (Ingold et al., 2018). The production of ferric iron (Fe^3+^) triggers the production of ROS and activates lipoxygenases, ultimately resulting in damage of cell membrane (Yang et al., 2016). Ferroptosis displayed unique morphological features and dense, compact mitochondria that distinguish from apoptosis and necrosis (Dixon et al., 2012). Biochemically, the solute carrier family 7 member 11-glutathione-glutathione peroxidase 4 (SLC7A11-GSH-GPX4) signaling axis is thought to comprise the major defense system in ferroptosis (Koppula et al., 2018). SLC7A11, which is also called xCT, serves as a critical constituent of the cystine/glutamate antiporter system Xc^−^ (Koppula et al., 2018). By facilitating the import of cystine and the export of glutamate in cellular, this antiporter activity relies on SLC7A11 (Koppula et al., 2018). Cysteine acts as the precursor that limits synthesis of GSH, a vital cofactor required by GPX4 to facilitate the detoxification of lipid peroxides (Koppula et al., 2021). SLC7A11, as a main BAP1 target gene in ferroptosis of cancers cell, is regulated by BRCA1-associated protein 1 (BAP1) on epigenetic mechanism (Zhang et al., 2018b). Specially, previous studies demonstrate that BAP1 downregulates SLC7A11 expression by reducing histone 2A ubiquitination occupancy on its promoter (Zhang et al., 2018b). As a result, cystine uptake is inhibited, resulting in increased lipid peroxidation and ultimately ferroptosis (Zhang et al., 2018b). Also, SLC7A11 and H2Aub location on its promoter are under the control of polycomb repressive complex 1 (PRC1), a key H2Aub ubiquitin ligase (Zhang et al., 2019). SLC7A11-regulted uptake of cystine and cysteine leads to activation of mTORC1 and subsequently promotes GPX4 protein biosynthesis via the Rag-mTORC1-4EBP pathway (Zhang et al., 2021). Furthermore, inactivation of mTORC1 makes cancer cells sensitive to ferroptosis by inhibiting GPX4 biosynthesis (Zhang et al., 2021). The process of ferroptosis is triggered by intracellular depletion of GSH and subsequent reduction in GPX4 activity. As a result, lipid peroxides cannot be efficiently regulated through the GPX4-catalyzed reaction, resulting in the accumulation of ROS. In the presence of Fe^2+^, lipids are oxidized in a Fenton-like way, further increasing ROS levels and ultimately inducing ferroptosis (Yang and Stockwell, 2008; Friedmann Angeli et al., 2014). Recently, a study termed a new cell death, named disulfidptosis (Liu et al., 2020; Liu et al., 2023). Under glucose starvation, disulfidptosis is observed in cancer cells, where SLC7A11 expresses abnormally, cystine uptake increases, cystine reduces to cysteine, and the NADPH pool depletes, which results in excess accumulation of intracellular disulfide molecules and rapid cell death (Liu et al., 2020; Liu et al., 2023).

The crucial endogenous mechanism of ferroptosis to counter lipid peroxidation is GPX4 (Chen et al., 2014; Matsuda et al., 2016). Glutathione serves as the primary co-ordinating substrate for cytosolic ferrous iron and is utilized by GPX4 as a precursor to elimination of the lipid peroxides subsequently inducing ferroptosis (Hider and Kong, 2011; Dixon and Stockwell, 2014; Friedmann Angeli et al., 2014; Yang et al., 2014). The effect of GPX4 depends on GSH produced by the plasma membrane X_c_^−^ (Dixon et al., 2012). SLC7A11 imports extracellular cystine into cells to promote GSH synthesis, resulting in protection from oxidative stress and ferroptosis (Zhang et al., 2021). Suppression of system Xc^−^-activity has a major impact on GSH synthesis by decreasing the intake of cystine, resulting in decrease of GPX activity and cell antioxidant capacity, ROS accumulation, and subsequently oxidative damage and ferroptosis (Li et al., 2020b).

In *C. elegans*, the increase of unstable ferric iron is correlated with genetic factors that expedite aging and pose a threat to lifespan (Xu et al., 2010; James et al., 2015; James et al., 2016) (Figure 3). In *C. elegans*, inhibiting ferroptosis by preventing lipid peroxidation and iron accumulation, reduce age-related cell death and significantly improves lifespan and healthspan (Jenkins et al., 2020). The increased threonine concentration diminished ferroptosis via ferritin-dependent way, activated ferritin 1 and antioxidant reaction, and extended longevity and health via a DAF-16 and HSF-1-dependent mechanism in *C. elegans* (Kim et al., 2022). Dietary lipids also can impact cell fates. In germ cells of *C. elegans*, ferroptosis is activated by dietary dihomo gamma linolenic acid (DGLA), which was modulated by redox reaction and iron metabolism (Perez et al., 2020). Endogenous ether-lipid synthesis and elevating monounsaturated fatty acid (MUFA) levels with genetic or dietary intervention are adequate to protect DGLA-induced germ-cell ferroptosis (Perez et al., 2020).

## Conclusion and future directions

For both human and worm, nutrition metabolism and cell death are the basis of life. In this review, we listed many crucial molecules and regulatory pathways in nematode and human. In *C. elegans*, haem cannot be biosynthesized by itself, so the multi-step enzymatic pathway of biosynthesizing haem is not conserved between human and worms. However, haem uptake and transport systems exist in cellular compartments in *C. elegans*, and haem binding proteins are evolutionarily conserved haem chaperone in yeast and mammalian cells. Vitamin and nutrient amino acid deficiency in both human and worms has negative effects on development and health. The crucial components of cell death are also conserved, such as EGL-1 and CED-family member (homolog of BCL-2 family, APAF-1 and Caspase in mammalian) in apoptosis, calpain proteases in necrosis, initiation of autophagy LET363 (homolog of mTOR) and autophagy modulatory transcription factor HLH-30 (homolog of TFEB). And ferroptosis also depends on iron overload, which further triggers the production of ROS and activities lipoxygenases in *C. elegans*. Nevertheless, many micronutrients and their metabolic pathway remain elusive. New types of cell death have been reported recently, like disulfidptosis. However, many novel cell death pathways remain undiscovered. *C. elegans*, as a power model, plays a crucial role for the basic research of nutrition metabolism and cell death.

Absorbing nutrients and processing cell death in nematode and human are related to survival. How does *C. elegans* intake micronutrients from environment, transport them to cell, and what are the details about enzymes and membrane carriers in these processes? What are the storage form, location, and utilization of micronutrients? The answers to these questions are still not clear. Finally, how can we apply better treatments for nutrition disorders? Similarly, the rigorous mechanism of cell death remains to be further explored. The probes for the identification research in cell death type rapidly need to be further exploited. The micronutrition metabolism and cell death, the genetic and functional characterization study about essential molecules and regulatory pathways in nematode is complex, which can still reveal the fundamental biology principles in human.
